# Case report: Two cases of multiple evanescent white dot syndrome with transient night blindness

**DOI:** 10.3389/fopht.2025.1557294

**Published:** 2025-02-17

**Authors:** Kanna Miyake, Mariko Egawa, Yoshinori Mitamura, Ryoji Yanai

**Affiliations:** Department of Ophthalmology, Tokushima University Graduate School of Biomedical Sciences, Tokushima, Japan

**Keywords:** electroretinography, multifocal electroretinography, multiple evanescent white dot syndrome, optical coherence tomography, transient night blindness

## Abstract

**Introduction:**

This study aimed to report two cases of multiple evanescent white dot syndrome (MEWDS) with transient night blindness.

**Case Presentation:**

Case 1: A 24-year-old man presented with acute visual loss and night blindness in his right eye. Examination revealed an enlarged blind spot and multiple white dots extending from the posterior pole to the peripheral retina in the right eye. Optical coherence tomography (OCT) revealed multiple disruptions of the ellipsoid zone (EZ). Full-field electroretinography (ffERG) demonstrated a more pronounced reduction in rod amplitude compared with cone amplitude in both eyes. After 3 months, the white dots, EZ disruption, and night blindness resolved spontaneously, and the ffERG amplitude normalized in the right eye. However, the enlarged blind spot persisted. Case 2: A 66-year-old woman presented with acute visual deterioration and night blindness in her right eye. The right eye exhibited an enlarged blind spot and numerous white spots widely extending from the posterior pole to the periphery. OCT revealed widespread EZ loss, and ffERG showed reduced rod and cone responses. SubTenon’s triamcinolone acetonide injection was administered, and 3 months after the injection, the night blindness, ffERG abnormalities, and EZ loss had resolved, but the enlarged blind spot remained.

**Conclusion:**

MEWDS rarely causes transient night blindness due to extensive rod dysfunction. However, outer retinal layer damage is reversible, with night blindness typically resolving within a few months.

## 1 Introduction

Multiple evanescent white dot syndrome (MEWDS) is a transient chorioretinopathy characterized by acute appearance of multiple white spots over a wide area of the fundus, which spontaneously resolve within a few months (1, 2). MEWDS occurs most commonly in young women with myopic vision and is usually unilateral. However, the cause of this disease is unknown, and immune response to infection or vaccines is suspected to be the cause. Patients often complain of visual abnormalities, such as foggy vision, photophobia, and blind spot enlargement in the acute phase, but night blindness is rare (0.7%) (3).

We report two cases of MEWDS with night blindness and marked rod-dominant amplitude reduction on full-field electroretinography (ffERG) from onset, with symptoms resolving within 3 months.

## 2 Case description

### 2.1 Case 1

A 24-year-old man was referred for blurred vision and unilateral night blindness in the right eye for 10 days. The patient had no history of cold symptoms, systemic illness, or ocular disease before the onset of symptoms. Best-corrected visual acuity (BCVA) was 0.7 × −5.5 diopters (D) in the right eye and 1.5 × −4.0D in the left eye.

**Figure 1:**
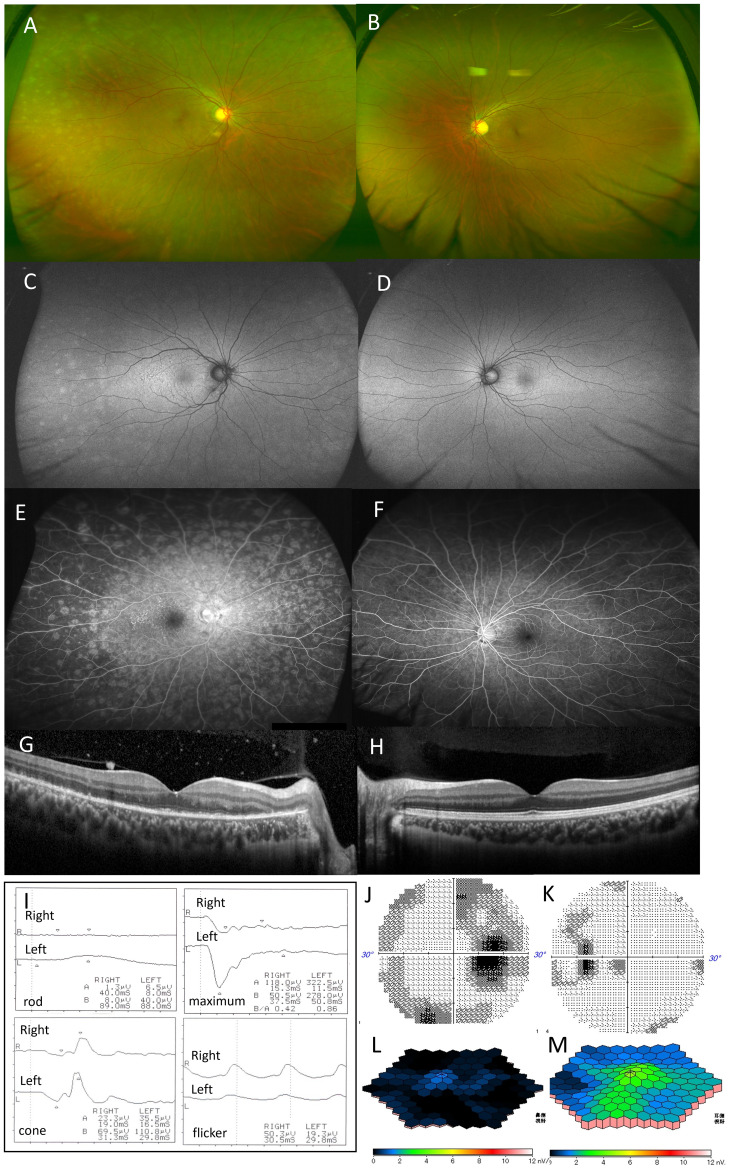
Case 1: A 29-year-old woman with multiple evanescent white dot syndrome in the right eye. Color fundus photography of the right eye revealed numerous white spots extending to the periphery of the fundus **(A)**. Fundus autofluorescence showed numerous faint hyperfluorescent spots in the periphery and diffuse hyperfluorescence in the posterior pole **(C)**. Fluorescein angiography showed numerous hyperfluorescent spots from the early to late stages **(E)**. Optical coherence tomography showed the extensive disappearance of the ellipsoid zone in the macula of the right eye and the disappearance of the outer nuclear layer on the temporal side of the optic disc **(G)**. No obvious abnormalities were found in the fundus of the left eye **(B, D, F, H)**. Full-field electroretinography (ERG) showed that the maximal ERG was negative-type in both eyes, more pronounced in the right, with reduced rod, cone, and flicker responses in both eyes **(I)**. Multifocal electroretinography revealed a marked amplitude reduction throughout the macula in the right eye **(J)**, with attenuation in the left eye, mainly near the optic nerve **(K)**. Humphrey visual field testing showed blind spot enlargement in both eyes, with an arcuate threshold depression in the right eye **(L, M)**.

Slit-lamp examination of the right eye revealed 2+ aqueous cells, 1+ vitreous cells, and numerous white dots extending from the macula to the periphery with granular changes in the fovea (**Figure 1A**). Fundus autofluorescence (FAF) showed multiple hyperfluorescent spots in the periphery, diffuse hyperfluorescence in the posterior pole, and mild hypofluorescence in the peripapillary area (**Figure 1C**). Fluorescein angiography (FA) revealed dense hyperfluorescent spots from early to late stages and optic disk leakage (**Figure 1E**). Indocyanine green angiography (ICGA)I showed small hypofluorescent spots. Optical coherence tomography (OCT) demonstrated extensive ellipsoid zone (EZ) disruption in the macula and outer nuclear layer (ONL) loss lateral to the optic disk in the right eye (**Figure 1G**).

The left eye showed no iritis, fundus white spots, or abnormalities on FAF, FA, ICGA, or OCT (**Figures 1B, D, F, H**). ffERG), performed per International Society for Clinical Electrophysiology of Vision standards with maximum pupillary dilation, revealed negative-type maximal electroretinograms in both eyes, with greater severity in the right eye. Rod responses were absent in the right eye and markedly reduced in the left eye; cone and flicker responses were also reduced bilaterally. Multifocal electroretinography (mfERG) showed a marked decrease in macular amplitude in the right eye and reduced amplitude primarily around the optic disk in the left eye (**Figures 1J, K**).

Humphrey Field Analyzer (HFA) 30-2 visual field testing revealed enlarged blind spots in both eyes and a bow-shaped threshold reduction in the right eye (**Figures 1L, M**). The right eye was diagnosed with MEWDS with night blindness, while the left eye was classified as asymptomatic MEWDS.

**Figure 2:**
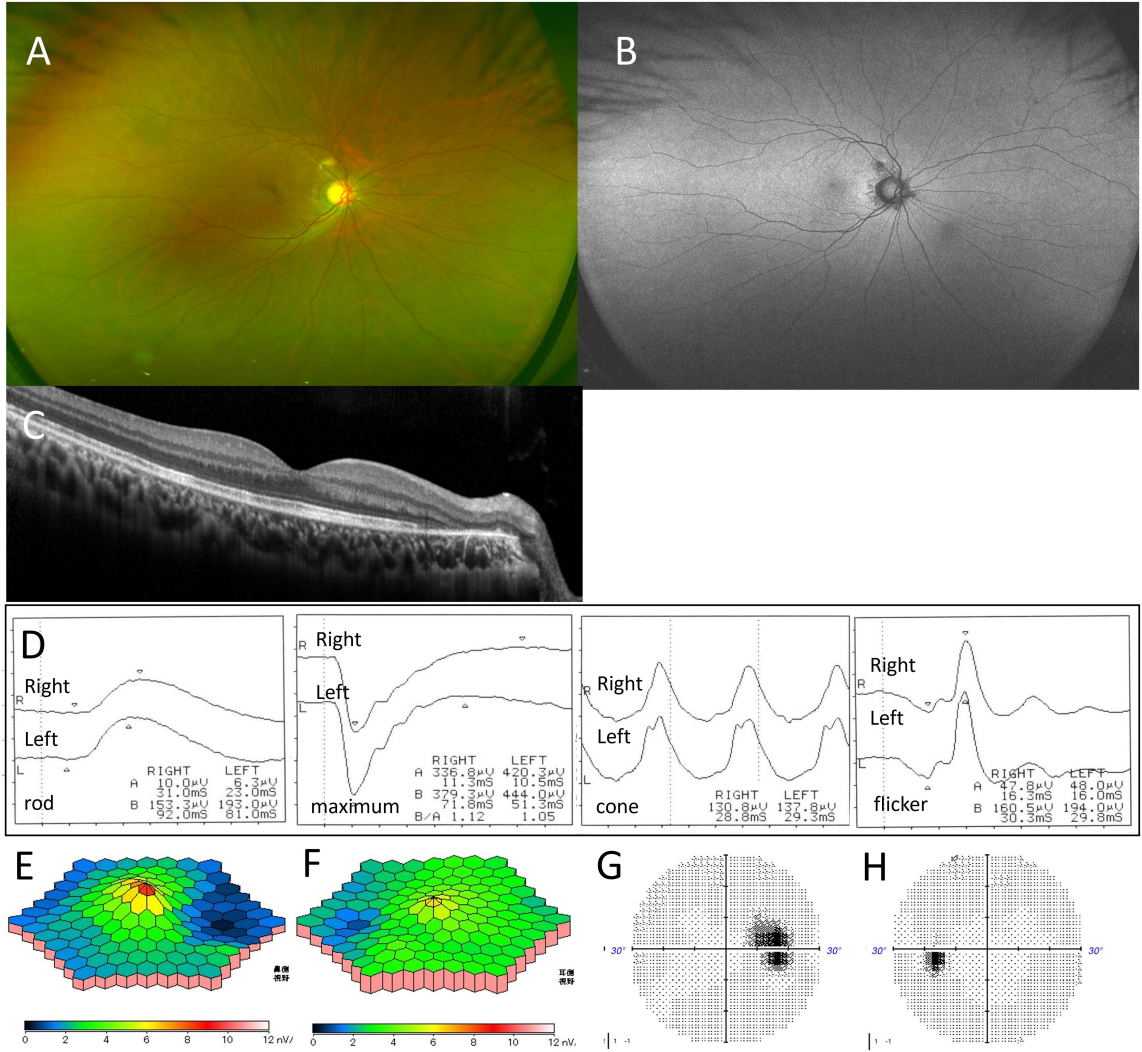
Case 1: A 29-year-old woman with multiple evanescent white dot syndrome in the right eye. After 3 months, color fundus photography showed that the white spots in the right fundus had disappeared **(A)**. Fundus autofluorescence indicated resolution of the hyperfluorescent spots, but hypofluorescence was observed due to peripapillary retinal atrophy **(B)**. Optical coherence tomography revealed recovery of the ellipsoid zone (EZ) in the right macula, though EZ loss and outer nuclear layer damage on the temporal side of the optic disc persisted **(C)**. Full-field electroretinography showed recovery of amplitude in both eyes **(D)**. Multifocal electroretinography revealed continued amplitude reduction on the optic disk side in the right eye **(E, F)**. Humphrey visual field testing showed that blind spot enlargement in the right eye remained **(G)**, while the visual field abnormality in the left eye had resolved **(H)**.

After 3 months without treatment, BCVA in the right eye improved to 1.5, and night blindness resolved. White spots and hyperfluorescent areas on FAF in the right fundus disappeared, but the peripapillary region showed irregular hypofluorescence due to chorioretinal atrophy (**Figures 2A, B**). OCT demonstrated recovery of the central macular EZ in the right eye, though EZ and ONL atrophy near the optic disk persisted (**Figure 2C**). ffERG responses normalized for maximal, rod, and cone amplitudes in both eyes, but mfERG in the right eye showed persistent amplitude reduction on the optic disk side (**Figures 2D–F**). The blind spot enlargement in the right eye remained, while visual field abnormalities in the left eye resolved (**Figures 2G, H**).

### 2.2 Case 2

**Figure 3:**
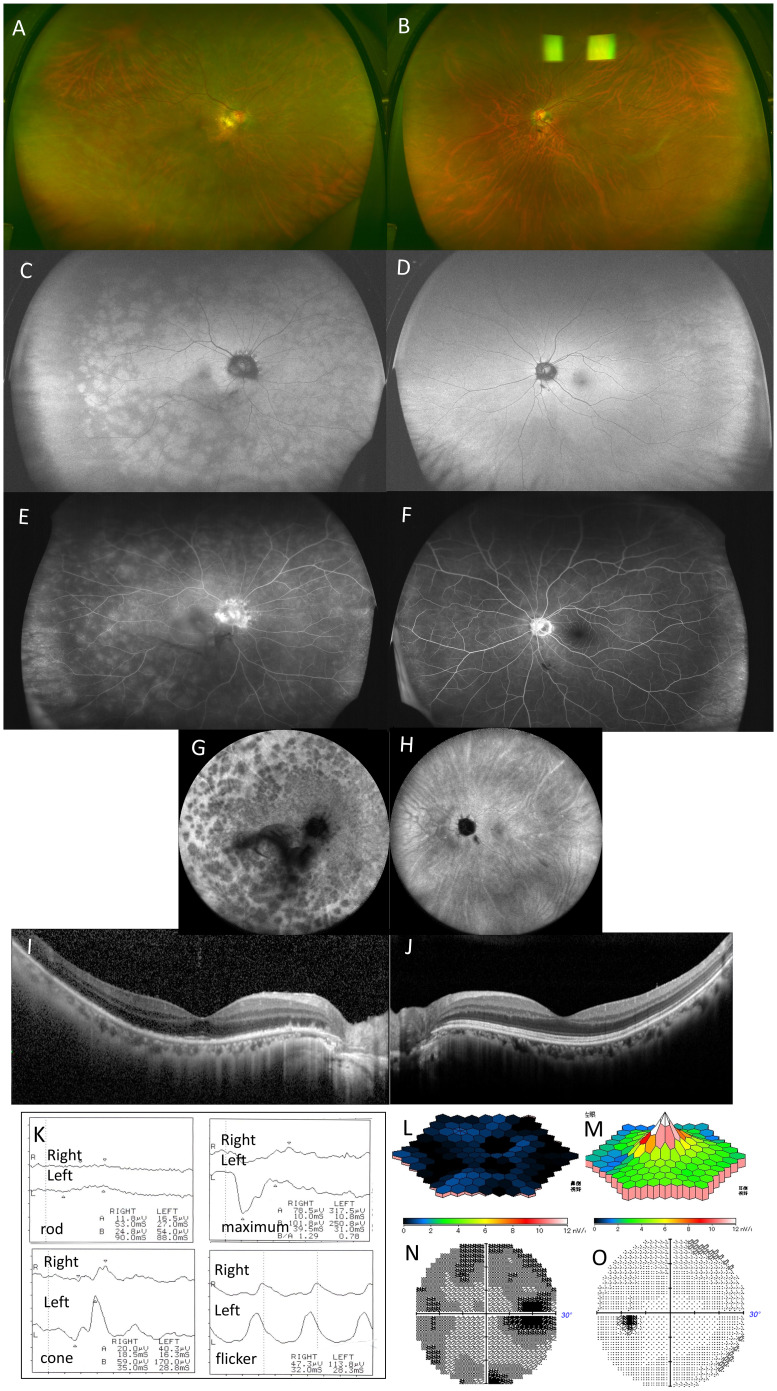
Case 2: A 66-year-old woman with multiple evanescent white dot syndrome in the right eye. Color fundus photography revealed numerous large white spots extending to the periphery of the right eye **(A)**. Fundus autofluorescence showed diffuse hyperfluorescence in the posterior pole and around the optic disc, with numerous large hyperautofluorescent patches extending to the periphery **(C)**. Fluorescein angiography revealed numerous hyperfluorescent spots **(E)**. Indocyanine green angiography showed numerous hypofluorescent spots in the late phase **(G)**. Optical coherence tomography revealed the disappearance of the ellipsoid zone throughout the entire macula in the right eye and thinning of the outer nuclear layer on the temporal side of the optic disc **(I)**. Full-field electroretinography (ERG) showed a subnormal maximal ERG in the right eye and a negative maximal ERG in the left eye, with absent rod responses in the right eye and reduced rod responses in the left. Cone responses and flicker ERG were reduced in the right eye **(K)**. Multifocal electroretinography showed a marked reduction in amplitude across the macula of the right eye **(L)**. Humphrey visual field testing showed an enlarged blind spot and overall threshold reduction in the right eye **(N)**. No white spots or abnormalities were found in the left eye, and no abnormal findings were observed in other tests **(B, D, F, H, J, M, O)**.

A 66-year-old woman presented with decreased vision and night blindness in the right eye for 1 week. She had no significant medical or ocular history. BCVA was 0.7 × −11.0 D in the right eye and 1.0 × −8.75 D in the left. Examination revealed 1+ aqueous cells, 1+ vitreous cells, and 1+ OCV in the right eye, with extensive white spots from the posterior pole to the peripheral area but no granular macular changes (**Figure 3A**). FAF of the right eye showed diffuse hyperfluorescence at the posterior pole and peripapillary area, protruding hypofluorescent areas from the optic disk, and large spotty hyperfluorescence extending to the periphery (**Figure 3C**). FA revealed mottled hyperfluorescence from early to late phases and optic disk leakage (**Figure 3E**), whereas ICGA showed numerous late-phase hypofluorescent spots, especially dense in the macula and peripapillary area (**Figure 3G**). OCT demonstrated widespread disruption of the EZ and ONL thinning around the optic disk (**Figure 3I**). ffERG of the right eye showed subnormal maximal ERGs, nearly absent rod response, and decreased cone and flicker amplitudes (**Figure 3K**). In the left eye, ffERG was negative with reduced rod amplitude (**Figure 3L**). mfERG showed an absent foveal response in the right eye (**Figure 3L**). Visual field testing revealed an enlarged blind spot and reduced macular threshold in the right eye (**Figure 3N**). The left eye had no white spots or abnormalities on FAF, FA, ICGA, OCT, mfERG, or visual field testing (**Figures 3B, D, F, H, J, M, O**).

**Figure 4:**
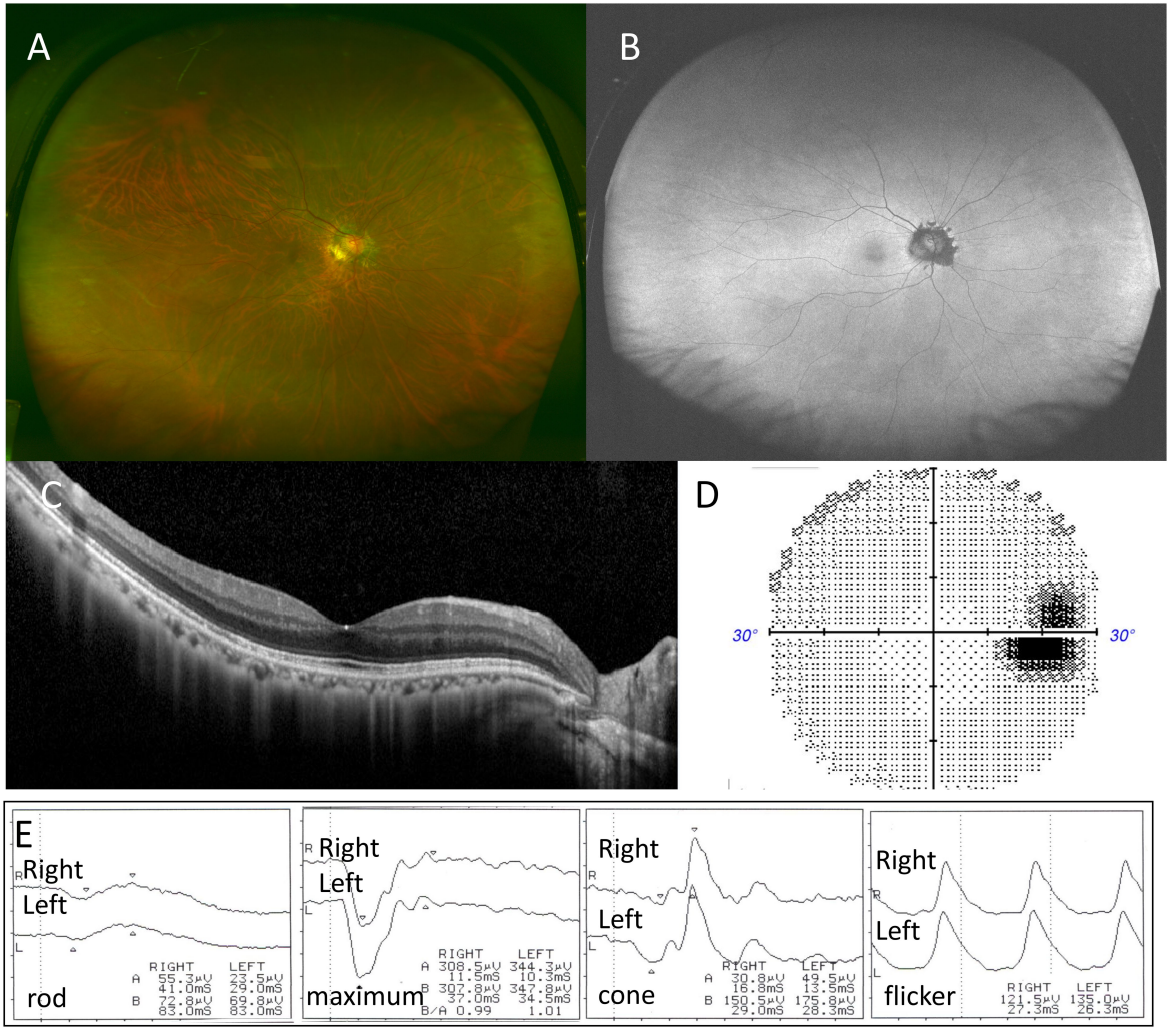
Case 2: A 66-year-old woman with multiple evanescent white dot syndrome in the right eye after 3 months. After 3 months, color fundus photography showed that the white spots on the right fundus had disappeared **(A)**, and fundus autofluorescence demonstrated resolution of the hyperfluorescent spots, with peripapillary protruding hypofluorescence remaining **(B)**. Optical coherence tomography revealed recovery of the ellipsoid zone (EZ) in the macula, but disruption of the EZ and thinning of the outer nuclear layer on the temporal side of the optic disc persisted **(C)**. Humphrey visual field testing showed residual blind spot enlargement **(D)**. Full-field electroretinography (ffERG) showed recovery **(E)**.

The patient was diagnosed with MEWDS with night blindness in the right eye and received a SubTenon’s triamcinolone acetonide injection. One month later, the white dots on the fundus became less prominent, and by two months, BCVA in the right eye improved to 1.2, with resolution of night blindness. Three months later, white spots on the right fundus (**Figure 4A**) and hyperfluorescent spots on FAF disappeared (**Figure 4B**), although peripapillary protruding hypofluorescence persisted (**Figure 4C**). OCT showed recovery of the macular EZ, but disruption of the EZ around the optic disk and ONL thinning remained. Residual blind spot enlargement persisted (**Figure 4D**), though ffERG showed near-complete recovery (**Figure 4E**).

## 3 Discussion

In the present case of MEWDS, significant rod damage was attributed to extensive outer retinal layer disruption extending to the periphery, causing acute night blindness. Consistent with the general course of MEWDS, night blindness resolved, and ERG amplitudes normalized within a few months. Retinitis pigmentosa, congenital stationary night blindness, and autoimmune retinopathy were excluded, as the symptoms were considered due to temporary photoreceptor dysfunction rather than photoreceptor cell loss.

Hashimoto et al. observed FAF findings in MEWDS and reported that lesions originate in the peripapillary and posterior pole, spread centrifugally to the periphery, and resolve from the periphery within approximately 3 months (4). In our cases, FAF images obtained using an ultrawide-field fundus camera showed hyperfluorescent spots extending to the periphery, even with vitreous opacity. These findings suggested extensive retinal pigment epithelium (RPE) damage. Moreover, the loss of rod response in ffERG indicates that peripheral rod function is severely impaired. These findings suggest that the night blindness was caused by dysfunction of the outer retina spreading to the periphery.

The ffERG in MEWDS typically shows reduced rod and cone responses during the acute phase, with recovery over several months (5, 6). Sieving et al. (5) reported predominant rod impairment, with rods becoming nondetectable in some cases; however, ffERG responses recovered within weeks. Kuniyoshi et al. (7) described a case with predominantly reduced cone responses in the acute phase; at 9 months, the fundus appeared normal, but the blind spot remained enlarged, and the EZ showed disruption, indicating residual cone damage. In the present cases, both rod and cone responses were reduced in ffERG during the acute phase, with a predominant rod reduction that recovered in 3 months. The rapid ffERG recovery indicates reversible photoreceptor damage.

Similarly, mfERG findings in MEWDS often show reduced cone amplitudes corresponding to affected lesions, with recovery within a few months. Moreover, it has been reported that mfERG amplitudes were supernormal in the early stages of MEWDS and returned to normal or decreased after 2 weeks of symptoms (8). However, in our cases, a significant reduction in mfERG responses was observed across the macula at the onset of the disease, with residual response reduction on the optic disk side persisting even after 3 months. Owing to severe inflammation in the posterior pole, the outer retinal damage, particularly around the optic disc, did not resolve, leading to peripapillary atrophy. Consequently, the enlarged blind spot remained. These findings suggest that when mfERG responses across the entire macula are markedly reduced at disease onset, there is a risk of irreversible outer retinal damage.

The multiple hypofluorescent spots seen in the late phase of ICGA in MEWDS often appear in areas without visible white spots on fundus examination, making ICGA a useful test to determine the full extent of the lesion. Two pathologies have been proposed to explain these hypofluorescent spots. One theory suggests nonperfusion or hypoperfusion of the choriocapillaris (9, 10), supported by laser speckle flowgraphy measurements showing reduced choroidal blood flow in the macula during MEWDS onset (11). This is thought to reflect mild, recoverable inflammation of the end-choriocapillary vessels, leaving no chorioretinal scars. Alternatively, it has been proposed that MEWDS involves primary photoreceptor-level inflammation, causing photoreceptor and RPE dysfunction, which temporarily impairs ICGA uptake by the RPE, resulting in hypofluorescent spots (12). The debate between these mechanisms—choroidal circulatory disturbance or photoreceptor inflammation—remains unresolved.

FAF, a noninvasive test reflecting outer retinal damage, especially in the RPE, has become a valuable diagnostic tool. In FAF, hyperfluorescence indicates RPE dysfunction with lipofuscin accumulation, while hypofluorescence signifies RPE atrophy or loss. In MEWDS, hypofluorescent spots on ICGA, reflecting RPE dysfunction, often coincide with hyperfluorescent spots on FAF and correlate with outer retinal layer dysfunction seen on OCT (9). In our cases, FAF showed hyperfluorescent spots extending to the periphery, diffuse hyperfluorescence in the posterior pole, and hypofluorescent areas surrounding the optic disk. These findings, combined with ICGA hypofluorescent spots, suggested significant circulatory disturbance centered on the optic disk, with widespread RPE dysfunction. Several months later, the hyperfluorescent spots disappeared, but peripapillary atrophy became more prominent, and hypofluorescence on FAF became distinct.

Widefield FAF proved useful in noninvasively identifying retinal damage undetectable by OCT or FA and tracking changes over time. Peripapillary atrophy has been reported in 23.5% of MEWDS cases, although less frequently than in acute zonal occult outer retinopathy (9, 13). Since MEWDS lesions originate near the papilla and may expand, severe inflammation involving the entire fundus can cause irreversible outer retinal layer damage, particularly in the peripapillary region. Early anti-inflammatory treatment may reduce the risk of residual visual field defects.

In Case 1, the fellow eye showed no abnormalities on OCT or FAF during the acute phase but had mild abnormalities on ffERG, mfERG, and visual field testing, all of which normalized after 3 months. In Case 2, the fellow eye lacked white spots or mfERG abnormalities but showed a reduced ffERG response, which also normalized at 3 months. While MEWDS is usually unilateral, binocular cases with mild second-eye inflammation have been reported (13). Li et al. noted that during the acute phase of MEWDS, many fellow eyes exhibited blind spot enlargement and peripheral visual field defects, often without OCT abnormalities. These visual field disturbances typically resolved during recovery (6). The 30 Hz flicker ERG response has been reported to be useful in detecting damage to inner retinal neurons (14). In Case 1, there were no specific subjective symptoms in the left eye, and no significant changes were observed on OCT. However, a decrease in the flicker ERG amplitude was noted, along with a slight decrease in the threshold in the central visual field on HFA. One possible explanation for the discrepancy between the flicker ERG response and subjective symptoms is that subclinical impairment of inner retinal neurons, undetectable by OCT, may account for the reduced flicker amplitude. These findings suggest that MEWDS may cause asymptomatic photoreceptor damage in the fellow eye during the acute phase, even when symptoms are limited to one eye.

MEWDS is prevalent among young women with myopia. A study of Japanese patients (age = 14–57 years [mean age = 29.9 years]) diagnosed with MEWDS reported predominance of high myopia (defined as >−6 D) in comparison to normal control participants (15). Myopia has been identified as a clinical feature of MEWDS, particularly within the Japanese population, indicating a marked propensity toward high myopia. This observation is consistent with the present case and suggests that elderly and male myopic eyes may also be susceptible to MEWDS. Punctate inner choroidopathy (PIC) predominantly affects young women with myopia and shares some clinical overlap with MEWDS (16). PIC is characterized by yellow–white lesions confined to the posterior pole without any anterior chamber or vitreous inflammation. OCT findings vary with disease progression but consistently demonstrate abnormalities at the inner choroid and RPE levels. Furthermore, FAF reveals hypofluorescent lesions, and ffERG results are typically normal. Despite the clinical resolution, persistent abnormalities detectable via OCT and FAF are common, and choroidal neovascularization develops in 40%–69% of cases. In the present cases, absence of corresponding findings led to the exclusion of PIC as a diagnosis.

In conclusion, MEWDS rarely causes night blindness, but when lesions extend to the peripheral fundus with significant rod impairment, night blindness may occur. However, rod function generally recovers, and night blindness resolves within a few months.

## Data Availability

The original contributions presented in the study are included in the article/supplementary material. Further inquiries can be directed to the corresponding author.
